# Augmented-reality-assisted minimally invasive quad-rod spinopelvic fixation for spinopelvic dissociation caused by pathological fracture due to extramedullary plasmacytoma: an evaluation of technique and its benefits

**DOI:** 10.1007/s00701-026-06818-2

**Published:** 2026-03-06

**Authors:** Rakesh Kumar, Kenneth T. Nguyen, Kento Yamanouchi, Venu M. Nemani, Jean-Christophe Leveque, Philip K. Louie, Rajiv K. Sethi

**Affiliations:** 1 Center for Neuroscience and Spine, Department of Neurosurgery, Virginia Mason Franciscan Health, Seattle, WA USA; 2 School of Medicine, Tulane University, New Orleans, LA USA; 3 Department of Orthopaedic Surgery, Keio University School of Medicine, Tokyo, Japan; 4 School of Public Health, University of Washington, Seattle, WA USA

**Keywords:** Augmented-reality, Spinopelvic fixation, Quad-rod construct, Spinopelvic dissociation, Plasma cell neoplasms, Plasmacytoma

## Abstract

Augmented reality (AR) provides real-time three-dimensional visualization for spinal instrumentation, though its role in spinopelvic stabilization remains limited. While AR-assisted pedicle and S2 alar-iliac screw placement has been reported, AR guidance for a dual-iliac quad-rod construct in spinopelvic dissociation has not. We describe AR-assisted minimally invasive placement of L4–L5 pedicle screws and bilateral iliac screws using intraoperative three-dimensional planning. Fixation was completed without complications and remained stable at last follow-up. This case suggests AR navigation may support accurate and efficient execution of complex minimally invasive spinopelvic fixation.

## Introduction

Plasma cell neoplasms arise from clonal plasma cell proliferation and present as solitary plasmacytomas or as part of multiple myeloma, occurring in bone or less commonly as extramedullary soft-tissue lesions. Sacral involvement poses unique structural, neural, and vascular challenges, although these tumors are typically radiosensitive with favorable responses to radiotherapy [6].

Surgical indications for treatment of plasma cell neoplasms include fracture fixation, decompressive laminectomy, and spine stabilization [1]. The preference for minimally invasive techniques arises from their capacity for minimal local tissue destruction, facilitating early postoperative recovery and the possibility of rapidly resuming radiation or chemotherapy for sacral lesions. In cases of bilateral sacral destructive lesions causing spinopelvic instability, iliac screws are a viable option for spinopelvic stabilization. Quad-rod augmentation with two independent sacroiliac anchoring points bilaterally is an effective stabilization technique, enhancing lumbosacral construct rigidity to promote fusion and reduce construct failure rates [7]. While augmented reality (AR) has been described for pedicle screw and S2 alar-iliac screw placement, its application to a minimally invasive dual-iliac quad-rod construct for spinopelvic dissociation has not been reported; integrating intraoperative real-time three-dimensional visualization may facilitate instrumentation within the narrow confines of the lumbosacral spine and iliac corridor.

## Case presentation

### History and presentation

**Fig. 1:**
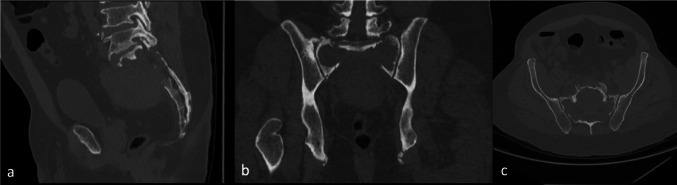
Preoperative CT spine (**A** sagittal; **B **coronal;** C **axial) mixed lytic destructive lesion involving bilateral sacral body with nondisplaced pathological fracture

**Fig. 2:**
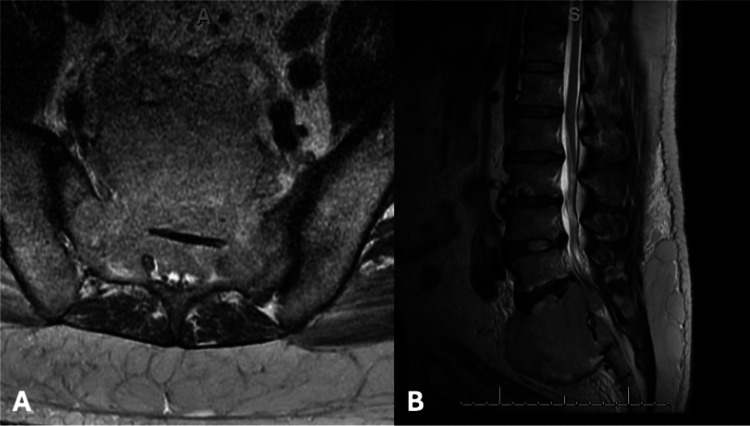
Preoperative MRI showing sacral extramedullary plasmacytoma on (**A**) axial and (**B**) sagittal T2-weighted images

A 63-year-old male with a known sacral extramedullary plasmacytoma presented with acute severe low back pain and bilateral buttock and thigh pain after five cycles of radiation therapy, resulting in inability to stand, sit, or ambulate, without bowel or bladder dysfunction or neurologic deficit. CT imaging demonstrated a destructive bilateral sacral body fracture with spinopelvic dissociation and interval progression compared with prior PET/CT (Fig. 1). A preoperative magnetic resonance imaging (MRI) study obtained one month prior to surgery demonstrated a destructive sacral lesion with associated marrow replacement and soft-tissue involvement consistent with extramedullary plasmacytoma (Fig. 2). Following a multidisciplinary team discussion, the patient underwent palliative augmented reality (AR) assisted minimally invasive percutaneous L4-Ilium instrumentation and spinopelvic fixation.

### Standard augmented reality registration and navigation workflow

**Fig. 3:**
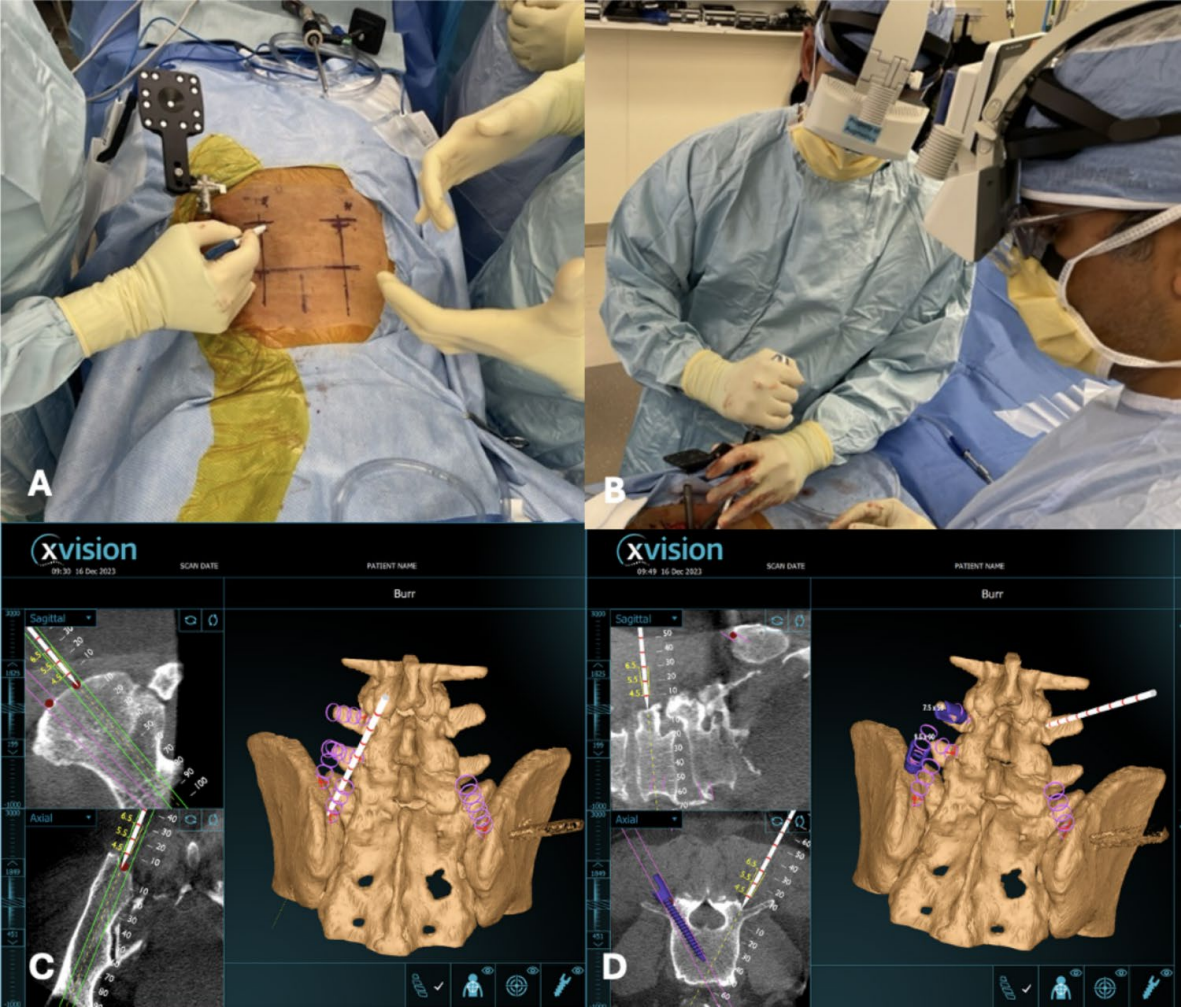
Augmented reality–assisted workflow showing (**A**) intraoperative setup with reference array and skin marking, **B** surgeons wearing the augmented reality head-mounted display, and screw trajectory planning with AR real-time 3D anatomy and navigation images in the sagittal and axial planes(**C**, **D**)

After patient positioning, a reference frame is rigidly secured to a long iliac pin. Intraoperative registration is performed by correlating the fixed reference marker with three-dimensional imaging, enabling real-time anatomical alignment. Registration accuracy is verified prior to instrumentation. The Augmedics head-mounted display (HMD) provides an augmented reality overlay of planned screw trajectories directly within the surgeon’s field of view (Fig. 3). Pedicle screw entry points are identified using AR guidance in conjunction with standard anatomical landmarks. Instrument advancement and trajectory are continuously monitored through the HMD, with conventional navigation views used for adjunctive confirmation.

### Operative technique

**Fig. 4:**
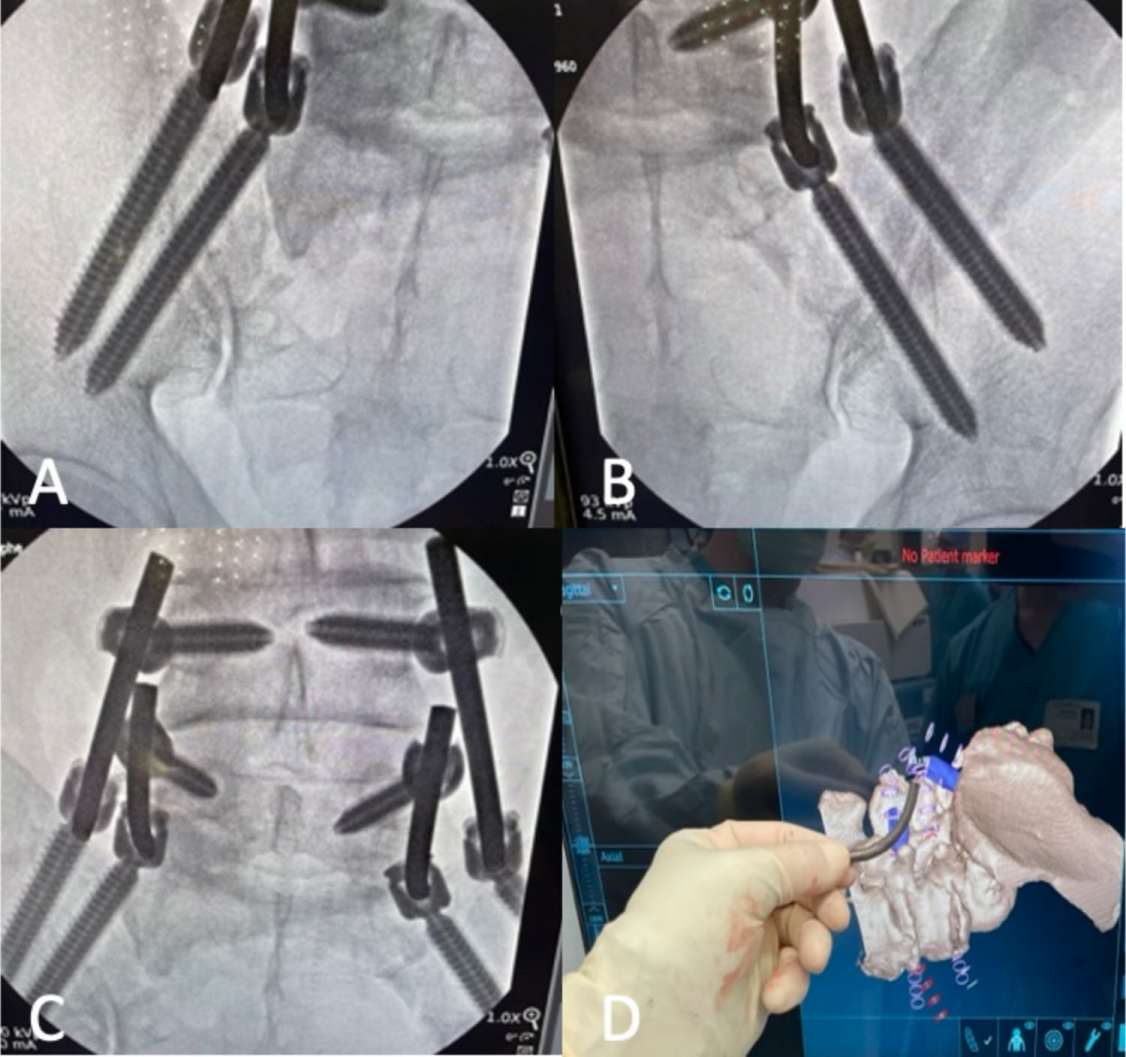
Intraoperative image of left and right iliac screws (**A**, **B**),Quad rod construct with one extending from L4 to an iliac bolt and the other stretching from L5 to a secondary iliac bolt (**C**), and rod contour planning using AR real time 3D images (**D**)

After informed consent, the patient underwent general anesthesia with neuromonitoring and was positioned prone on a Jackson frame. The lumbosacral region was prepped and draped to the posterior superior iliac spine, a right iliac reference array and AR fiducials were secured, and an intraoperative CT scan was obtained. Using augmented-reality-based head-mounted displays (Augmedics, Arlington Heights, IL, USA), a 3D AR system was employed to plan two 4.5 cm skin incisions, positioned off the midline and centered over the L4 transverse processes and extending through the medial portion of the iliac crest. Utilizing real-time 3D navigation, entry points and trajectories were planned for bilateral L4, L5, and iliac screws, as extensive sacral tumor involvement precluded S2 alar-iliac fixation (Fig. 4). Bilateral 6.5 × 45 mm L4 and L5 pedicle screws were successfully placed. The augmented reality system was used to plan and navigate two K-wires into each iliac wings and 4 total iliac screws were placed, each measuring 9.5 mm x 90 mm. Cobalt chrome rods were then fashioned and positioned, with one rod on each side extending from L4 to the dorsal/lateral iliac bolt and the other stretching from L5 to a secondary more ventral/medial iliac bolt (Fig. 4). This staggered quad-rod configuration (two rods terminating at L4 and two at L5) was selected to function as four independent fixation points, improving load distribution across the lumbosacral junction while simplifying rod passage and alignment under minimally invasive constraints. The procedure was completed with an estimated blood loss of 100 mL and an operative time of approximately 2.5 h, including the time required for intraoperative CT acquisition and planning.

### Postoperative course

**Fig. 5:**
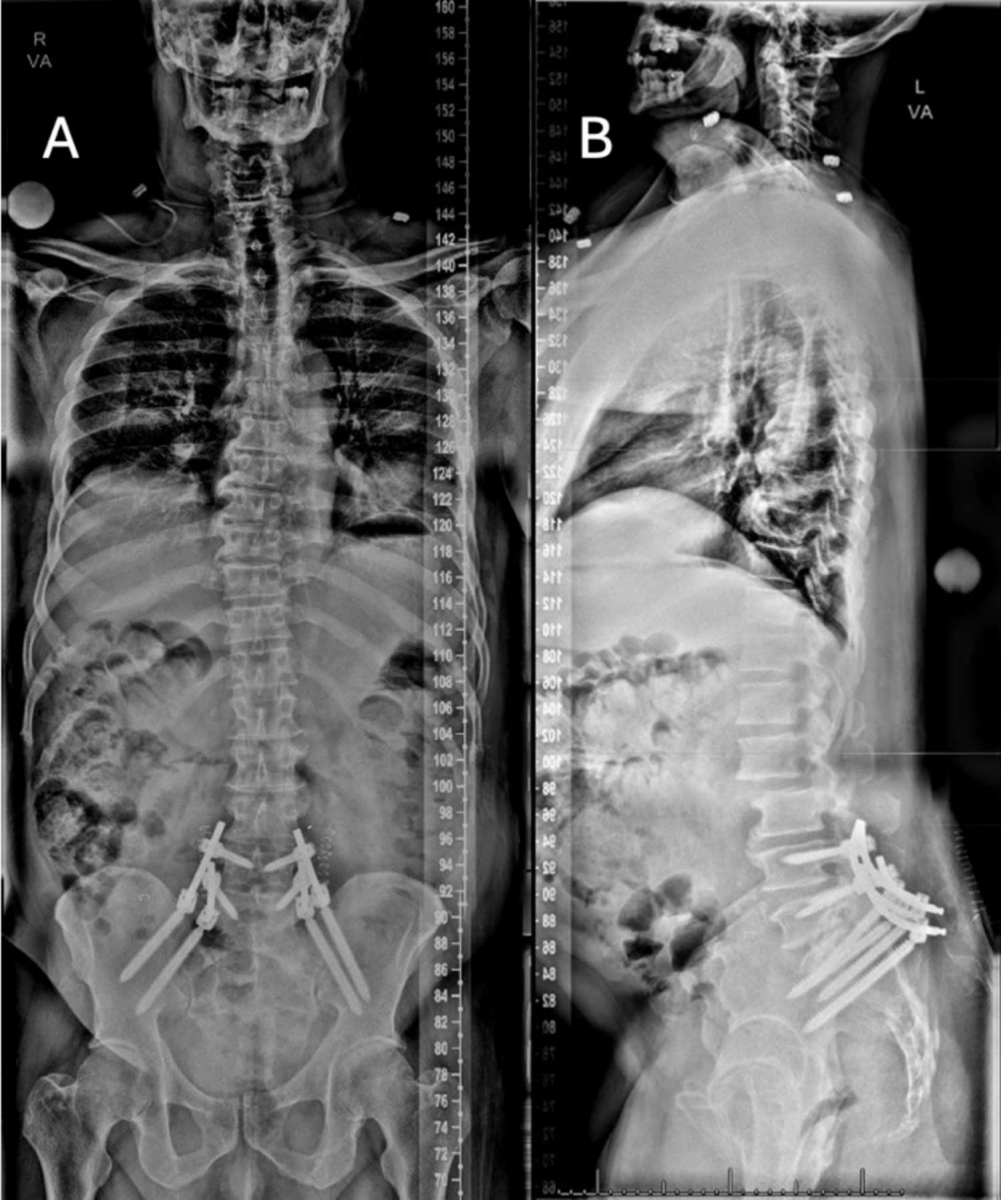
Postoperative upright X-ray. **A** AP view. **B** Lateral view (note that this view includes a stitching error between the L2 and L3 vertebral bodies that does not affect visualization of the fusion construct)

No complications were observed in the postoperative period. The patient regained ambulation on the first postoperative day. Upright spinal films obtained postoperatively confirmed the accurate placement of instrumentation (Fig. 5). The patient’s preoperative symptoms were attributed to sacral pain from spinopelvic dissociation rather than true radiculopathy. By postoperative day 1, he reported marked pain improvement and was able to stand, and by postoperative day 4 he was ambulating with physical therapy with tolerable pain. Two weeks after surgery the patient resumed palliative radiation therapy. At 18-month follow-up, clinic notes documented improved hip pain with decreased opioid requirements, and CT and MRI demonstrated stable instrumentation without loosening or failure.

## Discussion

Iliac screw fixation establishes a caudal anchor point that provides enhanced biomechanical force for fusion, an increased pullout strength, and excellent stability across the lumbosacral junction compared to sacral fixation alone [5]. In situations involving lytic sacral tumors affecting the posterior and posterolateral aspects of the sacrum where S2 alar-iliac (S2AI) fixation may not be feasible the use of iliac screws becomes essential [5].

The utilization of minimally invasive iliac screw fixation with navigation or robotic guidance represents a promising technique increasingly employed in operations addressing deformities and spinal lesions. Augmented Reality (AR) presents an integrated environment, merging computer-generated images into a real stereotaxic space, enhancing awareness of real-world depth perception. In augmented reality surgical navigation (ARSN) systems relying on optical tracking, planned trajectories are overlaid directly within the surgeon’s line of sight, allowing continuous visualization of the operative field without diverting attention to external screens and offering ergonomic advantages compared with fluoroscopy- or robot-based workflows. AR-assistance placement of instrumentation is an emerging technology in the field of spinal surgery [2].

In the context of oncologic patients, the utilization of minimally invasive AR assistance offers a safe and precise method for positioning iliac screws. This minimally invasive approach minimizes soft tissue dissection and may reduce postoperative complications such as infection, which holds particular significance in cancer-related cases. Given the potential complexities of concurrent oncologic treatments in the postoperative course, the ability to perform operations with minimal soft tissue disruption allows for rapid healing and early resumption of palliative postoperative radiation therapy and chemotherapy, which may enhance overall treatment outcomes [4].

The working area for instrumentation placement in the lumbosacral spine is relatively limited given converging screw trajectories and the complex three-dimensional relationship between the lordotic lower lumbar spine, the sacrum and the ilium/iliac wings. This case report delves into the innovative application of augmented reality (AR) in the realm of spine surgery, specifically focusing on a minimal invasive quad-rod spino-pelvic fixation. While limited reports support the enhanced accuracy, reduced need for screw revision, and diminished radiation exposure for AR-assisted S2AI screw placement [3], there is a scarcity of discussions on AR-navigated iliac screw fixation in the existing literature. This report marks the first instance of AR-assisted quad rod fixation utilizing iliac screw placement. In this initial experience with four AR-assisted minimally invasive iliac screws, there were no intraoperative complications, no new neurologic deficits, no requirement for screw revision, and no evidence of early hardware failure. Compared to open techniques for the same instrumentation construct, the blood loss of 100 mL is noteworthy. Moreover, the reduced surgical footprint and tissue manipulation may enable patients to undergo radiation earlier with a reduced risk of wound complications.

## Conclusion

This report describes the use of augmented reality (AR) to guide minimally invasive iliac screw placement for quad-rod spinopelvic fixation in the setting of destructive sacral metastatic disease. While prior literature has largely focused on AR-assisted S2 alar-iliac fixation, this case demonstrates the technical feasibility of AR-guided iliac fixation with short-term clinical and radiographic stability. No intraoperative complications, new neurologic deficits, screw revision, or early hardware failure were observed. In addition, AR navigation provided real-time, line-of-sight visualization of planned trajectories within the operative field, allowing continuous surgeon focus and potentially improved ergonomics compared with workflows requiring external displays. These potential workflow advantages warrant further study to define the role of AR navigation in complex oncologic spinopelvic fixation.

## Data Availability

No datasets were generated or analysed during the current study.
